# Synergistic effect of PAK and Hippo pathway inhibitor combination in NF2-deficient Schwannoma

**DOI:** 10.1371/journal.pone.0305121

**Published:** 2024-07-31

**Authors:** Dorothy Benton, Hoi Yee Chow, Sofiia Karchugina, Jonathan Chernoff

**Affiliations:** 1 Department of Biochemistry & Molecular Biology, Drexel University College of Medicine, Philadelphia, Pennsylvania, United States of America; 2 Cancer Signaling and Microenvironment Program, Fox Chase Cancer Center, Philadelphia, Pennsylvania, United States of America; NCMLS, Radboud University Nijmegen Medical Center, NETHERLANDS, KINGDOM OF THE

## Abstract

Neurofibromatosis type 2 is a genetic disorder that results in the formation and progressive growth of schwannomas, ependymomas, and/or meningiomas. The NF2 gene encodes the Merlin protein, which links cell cortical elements to the actin cytoskeleton and regulates a number of key enzymes including Group I p21-activated kinases (PAKs), the Hippo-pathway kinase LATS, and mTORC. While PAK1 and PAK2 directly bind Merlin and transmit proliferation and survival signals when Merlin is mutated or absent, inhibition of Group 1 PAKs alone has not proven sufficient to completely stop the growth of NF2-deficient meningiomas or schwannomas *in vivo*, suggesting the need for a second pathway inhibitor. As the Hippo pathway is also activated in NF2-deficient cells, several inhibitors of the Hippo pathway have recently been developed in the form of YAP-TEAD binding inhibitors. These inhibitors prevent activation of pro-proliferation and anti-apoptotic Hippo pathway effectors. In this study, we show that PAK inhibition slows cell proliferation while TEAD inhibition promotes apoptotic cell death. Finally, we demonstrate the efficacy of PAK and TEAD inhibitor combinations in several NF2-deficient Schwannoma cell lines.

## Introduction

Neurofibromatosis type 2 (NF2) is an autosomal dominant tumor predisposition syndrome characterized by the development of multiple tumors in the central nervous system. NF2 patients are likely to develop Schwannomas on the eighth cranial nerve that are followed by progressive deafness and spinal cord compression as well as meningiomas and peripheral nerve tumors. Effective treatment for these patients is lacking, with risky surgery being the most reliable approach and pharmacotherapies having inadequate results. The hallmark mutation of this condition is inactivation or deletion of the *NF2* gene, which codes for the protein Merlin. Merlin does not have enzymatic activity, is usually absent in NF2-related tumors, and does not present a ready target for manipulation by small molecules. For these reasons, attempts have been made to target signaling proteins whose activities increase when Merlin function is lost. Among the signaling pathways regulated by Merlin, three in particular—PI3K/AKT/mTORC, Hippo, and Group I PAKs (PAK1, -2, and -3, which directly bind to and are inhibited by Merlin)—have emerged as lead candidates for therapeutic intervention [1]. Unfortunately, to date the mTORC1 inhibitor everolimus and the combined mTORC1/2 inhibitor vistusertib, respectively, have failed to provide convincing clinical benefit in human trials [2,3]. Loss of PAK function, in particular the PAK1 isoform, is associated with slower growth of NF2-deficient meningioma and schwannoma cells and has been proposed as a plausible therapeutic target for these tumors [4–6]. However, the development of PAK inhibitors has been hampered by the requirement for PAK2 in normal cardiovascular function in adult animals [7].

The Hippo pathway is a highly conserved signaling network that was first discovered in a *Drosophila melanogaster* genetic screen where Hippo gene mutations cause overgrowth of organs and tissues [8,9]. The pathway plays a key role in embryonic development, organ size determination, tissue regeneration, and wound healing [10]. In cancer, the pathway poses a unique challenge as the kinases that make up the cascade, which would be more straightforward to target, are tumor suppressors. The canonical Hippo pathway is made up of kinases MST1/2, with scaffolding protein SAV1, and LATS1/2, with scaffolding protein MOB1. This kinase cascade ultimately phosphorylates transcriptional coactivators YAP and TAZ. When phosphorylated, YAP and TAZ are sequestered in the cytoplasm by 14-3-3 proteins and targeted for degradation. When unphosphorylated, YAP and TAZ enter the nucleus where they bind to transcription factors TEAD1-4. This binding promotes transcription of Hippo target genes, which generally promote tumor initiation and progression and resistance to apoptosis [10]. Specifically, YAP/TAZ upregulate the anti-apoptotic BCL-2 and IAP family members, suppressing mitochondrial induced apoptosis [11].

Targeting the Hippo pathway has been challenging, but the recent development of TEAD palmitoylation inhibitors has made it more feasible [12]. TEAD palmitoylation is necessary for binding to YAP/TAZ and subsequent transcription of Hippo targets. By blocking this post translational modification, TEAD inhibitors are able to prevent YAP-TEAD binding as well as transcription of Hippo target genes. TEAD palmitoylation inhibitors like those developed by Vivace Therapeutics were first tested in NF2-deficient mesothelioma and have also been effective in reducing Schwannoma tumor size *in vivo* without side effects [13,14].

Combination therapy is a strategy that uses multiple drugs, typically at lower doses than are used in monotherapy. In modern cancer treatment, many patients are treated with multiple drugs. Synergy is a phenomenon where the total effect of two drugs is greater than the sum of each alone. Synergy allows for drugs to be used at lower doses, minimizing side effects. In this study, we demonstrate the efficacy of Group I PAK inhibition and TEAD-YAP binding inhibition in NF2-deficient schwannoma cells. The combination of PAK and TEAD inhibition is synergistic and may be a potential option for more effective treatment for NF2 syndrome.

## Methods

### Drugs

IK-930, a selective, small molecule palmitate inhibitor of TEAD that disrupts TEAD-dependent transcription, was provided by Ikena Oncology. TED-347 (HY-125269), an irreversible, covalent and allosteric inhibitor of the TEAD and YAP protein-protein interaction, FRAX-1036 (HY-19538), a group I PAK inhibitor, G-5555 (HY-19635), a group I PAK inhibitor, and NVS-PAK1-1 (HY-100519), a PAK1-specific inhibitor, were purchased from MedChemExpress. NSC682769, a benzazepine compound that binds to YAP and prevents YAP-TEAD interaction, was obtained from the NCI Developmental Therapeutics Program (DTP).

### Cell culture

HEI-193 (NF2 mutant) human schwannoma cells and SC4 (NF2-null) mouse schwannoma cells were generous gifts from Dr. Marco Giovannini (House Ear Institute, CA). Human Schwann cells (NF2- null and WT) were a generous gift of Dr. Joseph Kissil [15]. HEI-193 and SC4 cells were cultured in high-glucose Dulbecco’s Modified Eagle Medium (DMEM) supplemented with 10% fetal bovine serum, 1 mM sodium pyruvate, 0.1 mM non-essential amino acids, and 100 U/ml penicillin/streptomycin at 37°C, 5% CO2. Human Schwann cells were cultured in high-glucose Dulbecco’s Modified Eagle Medium (DMEM) supplemented with 10% fetal bovine serum, 1 mM sodium pyruvate, and 100 U/ml penicillin/ streptomycin at 37°C, 5% CO2.

### Viability assays

Cells were plated in white 96-well plates at 1000 cells per well and left overnight. For experiments carried out at 0.5% FBS, cells were washed with PBS and DMEM containing 1 mM sodium pyruvate, 0.1 mM non-essential amino acids, and 0.5% FBS was added and left overnight. The next day, drugs were added and cells were left for the indicated times. Viability was determined by CellTiter-Glo (Promega) according to the manufacturer’s instructions. For nuclei counts, cells were stained with Hoechst 33342 (ThermoFisher) according to the manufacturer’s instructions and incubated for 1 hour and imaged with an ImageXpress Micro Confocal.

### Immunoblotting

Cells were lysed with RIPA buffer (150mM sodium chloride, 50mM Tris-HCl, 1% Nonidet P-40, 0.5% sodium deoxycholate, 0.1% SDS) for 20 minutes on ice. Samples were separated on a 4–20% gel and transferred onto polyvinylidene difluoride (PVDF) membranes by electrophoresis. Membranes were blocked with Tris-buffered saline plus 0.1% Tween 20 (TBST) containing 5% milk powder for 1 hour at room temperature before being incubated overnight at 4°C with primary antibodies. Antibodies for PAK1 (#2606), PAK2 (#2608), Phospho-PAK1 (Ser144)/PAK2 (Ser141) (#2606), YAP1 (#4912), Merlin (#6995), GAPDH (#2118), and β-actin (#4970) were purchased from Cell Signaling Technologies. Blots were incubated with a horseradish peroxidase (HRP)-conjugated secondary antibody for 1 hour at room temperature and visualized with enhanced chemiluminescence (ECL) reagents on a FluorChem E System (ProteinSimple).

### Gene knockdown

HEI-193 cells were transfected with FlexiTube siRNA for YAP1 (S102662954) purchased from Qiagen using Lipofectamine RNAiMAX Transfection Reagent (ThermoFisher) according to the manufacturer’s instructions. Cells were plated in 24-well plates for 24 hours. RNA (5pmol/well) and Lipofectamine reagent (1.5 mL/well) were combined and incubated for 5 minutes at room temperature. The siRNA-lipid complex was then added to cells.

### qPCR

Cells were grown in 6-cm plates and treated with indicated drugs. Total RNA was extracted using RNeasy Minikit (Qiagen) according to manufacturer’s instructions. Cells were lysed and lysate was filtered. Ethanol was added to the homogenized lysate and mixed thoroughly. Lysate was passed through the RNA column and a DNase reaction mixture was added to digest the DNA. The column was washed and dried and RNA was eluted with 60 μL of RNase-free-H_2_O. ProtoScript II First Strand cDNA Synthesis Kit (NEB E650S) was used to produce cDNA. The template RNA, random primers, and enzyme mix were added to the reaction mixture before incubating at 42°C for one hour. The enzyme mix was inactivated at 80°C for 5 minutes. Template cDNA, forward and reverse primers, and SYBR Green mastermix were combined for each sample. Quantitative RT-PCR reactions were performed using FastStart Universal SYBR Green Master (ROX) (Roche) and PrimeTime qPCR primers (IDT) using QuantStudio 6 Pro system. Results were analyzed using Data & Analysis software 2.6.0. (Applied Biosystems).

### Xcelligence

The effect of YAP knockdown on HEI-193 cell proliferation was assessed using the xCELLigence Real-Time Cell Analyzer Dual Plate (RTCA-DP) system according to the manufacturer’s recommendations (Acea, Biosciences Inc., San Diego, CA, USA). Briefly, HEI-193 cells were cultured in a 16-well E-plate (ACEA Biosciences Inc., San Diego, CA, USA) for 24 hours. Cells were transfected with siRNA. The cell index values were monitored every hour for 64 hours.

### Colony formation assay

Cells were plated in 6-well plates 1000 cells per well. Indicated compounds were added to media and cells were grown for 10–14 days. Cells were washed with PBS and treated with 6% glutaraldehyde and 0.5% crystal violet. Cells were washed three times with water and left to dry. Plates were photographed and colonies were counted.

### Proliferation

Cells were plated in 6-well plates and treated with indicated drugs for 24 hours. 10μM EdU was added to cells for 2 hours before collection. Cells were fixed, permeabilized, and detected using Click-iT Plus EdU Flow Cytometry Assay Kit Alexa Fluor 647 picolyl azide (Invitrogen) according to manufacturer’s instructions. Briefly, cells were washed in 1% BSA in PBS. Cells were pelleted and resuspended in fixative before 15 minutes of incubation at room temperature. Cells were then washed in 1% BSA in PBS, pelleted, and resuspended in a saponin-based permeabilization and wash reagent. The fluorescent dye picolyl azide and other buffer additives were added to cells and cells were incubated for 30 minutes at room temperature protected from light. Finally, cells were washed with wash buffer and resuspended in assay buffer for FACS analysis. Fluorescence (active caspase activity) was measured on a FACSymphony A5 cytometer (BD Biosciences).

### Cell cycle

Cells were plated in 6-well plates and treated with indicated drugs for 24 hours. Cells were lifted from plates with 0.45% Trypsin with EDTA, fixed with 70% ethanol dropwise while vertexing, and left on ice for 30 minutes. Cells were treated with 100μg/ml RNase A and PI solution. Fluorescence was measured on a FACSymphony A5 cytometer (BD Biosciences). Cell cycle phases were determined using the Watson Pragmatic model via FlowJo [16].

### Live/Dead

Cells were plated in 6-well plates and treated with indicated drugs for 24 hours. Cells were lifted from plates with 0.45% Trypsin with EDTA. Cells were treated with LIVE/DEAD™ Viability/Cytotoxicity Kit for mammalian cells (Invitrogen) according to the manufacturer’s instructions. Cells were washed and resuspended in PBS and 2 μL of 50 μM calcein AM working solution and 4 μL of 2 mM ethidium homodimer-1 stock was added to each milliliter of cells. Cells were left to incubate for 15–20 minutes. Live cells (calcein AM positive, ethidium homodimer negative) and dead cell populations (calcein AM negative, ethidium homodimer positive) were determined with a FACSymphony A5 cytometer (BD Biosciences).

### Caspase activity

Cells were plated in 6-well plates and left overnight. The next day, cells were treated with indicated drugs for 24 hours. Caspase activity was determined with Generic Caspase Activity Assay Kit (Fluorometric—Green) (ab112130) (Abcam). Briefly, TF2-VAD-FMK was added to media and incubated for 2 hours. Cells were collected, washed in PBS, and resuspended in assay buffer. Fluorescence was measured on a FACSymphony A5 cytometer (BD Biosciences).

### Statistical analysis

All experiments were performed at least three times. Results were reported as means ± standard deviation (SD). The significance of the data was determined by two-tailed, unpaired Student’s *t-*test with *P* < 0.05 considered statistically significant.

## Results

**Fig 1:**
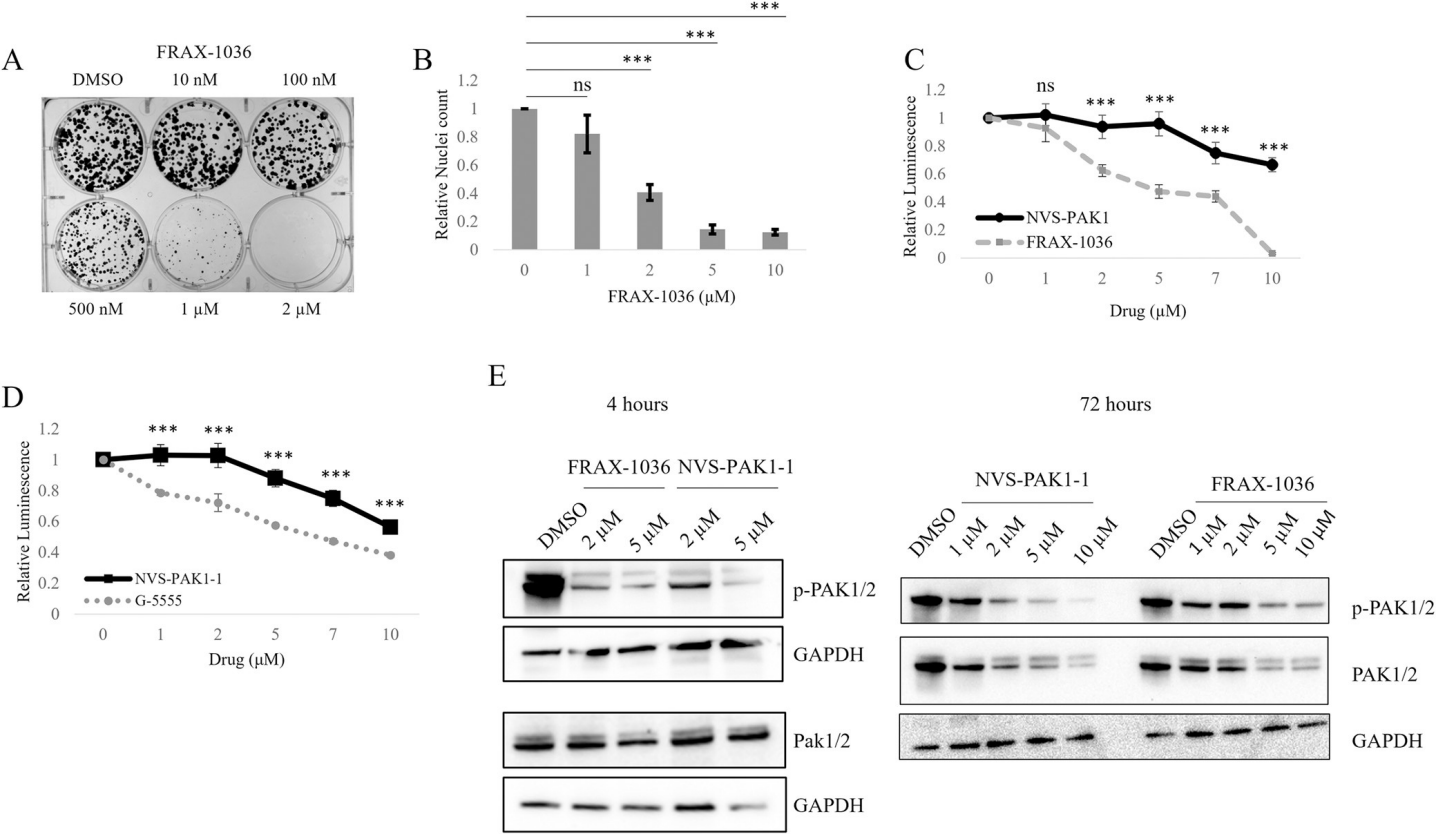
Group I PAK inhibition reduces cell growth in human NF2-deficientschwannoma. Colony formation assays were performed to assess the efficacy of FRAX-1036 (A). Nuclei counts were performed on HEI-193 cells treated with FRAX-1036 for 72 hours (B). Cell viability was determined by CellTiter-Glo for HEI-193 cells treated with NVS-PAK1-1 and FRAX-1036 (C) or G-5555 (D) for 72 hours. Immunoblot analysis showed reduction in phospho-PAK1/2 (S144/S141) when treated with PAK inhibitors for four or 72 hours (E). Error bars depict SD. ns = not significant; ** = p<0.01; *** = p<0.001.

As has been previously tested, Group I PAK inhibition is successful in perturbing schwannoma cells in models of NF2 syndrome [5,17]. To confirm these findings, we treated a human schwannoma cell line (HEI-193) that produces a truncated and inactive Merlin protein with the Group I PAK inhibitor FRAX-1036. The drug reduced colony formation of the cells (Fig 1A). It also significantly reduced cell number, by nuclei count, over 72 hours (Fig 1B). While FRAX-1036 has been successfully tested in other NF2-deficient cell types, inhibiting PAK2 *in vivo* has been shown to be associated with on-target cardiovascular toxicities [7]. In an attempt to overcome this obstacle, we compared a PAK1-specific inhibitor, NVS-PAK1-1, to the pan-group I PAK inhibitors FRAX-1036 and G-5555 (Fig 1C and 1D). However, both G-5555 and FRAX-1036 reduced cell viability significantly better than PAK1-specific NVS-PAK1-1, suggesting that Group 1 PAKs may have redundant functions in NF2-deficient Schwann cells, requiring a pan-Group 1 PAK inhibitor.

**Fig 2:**
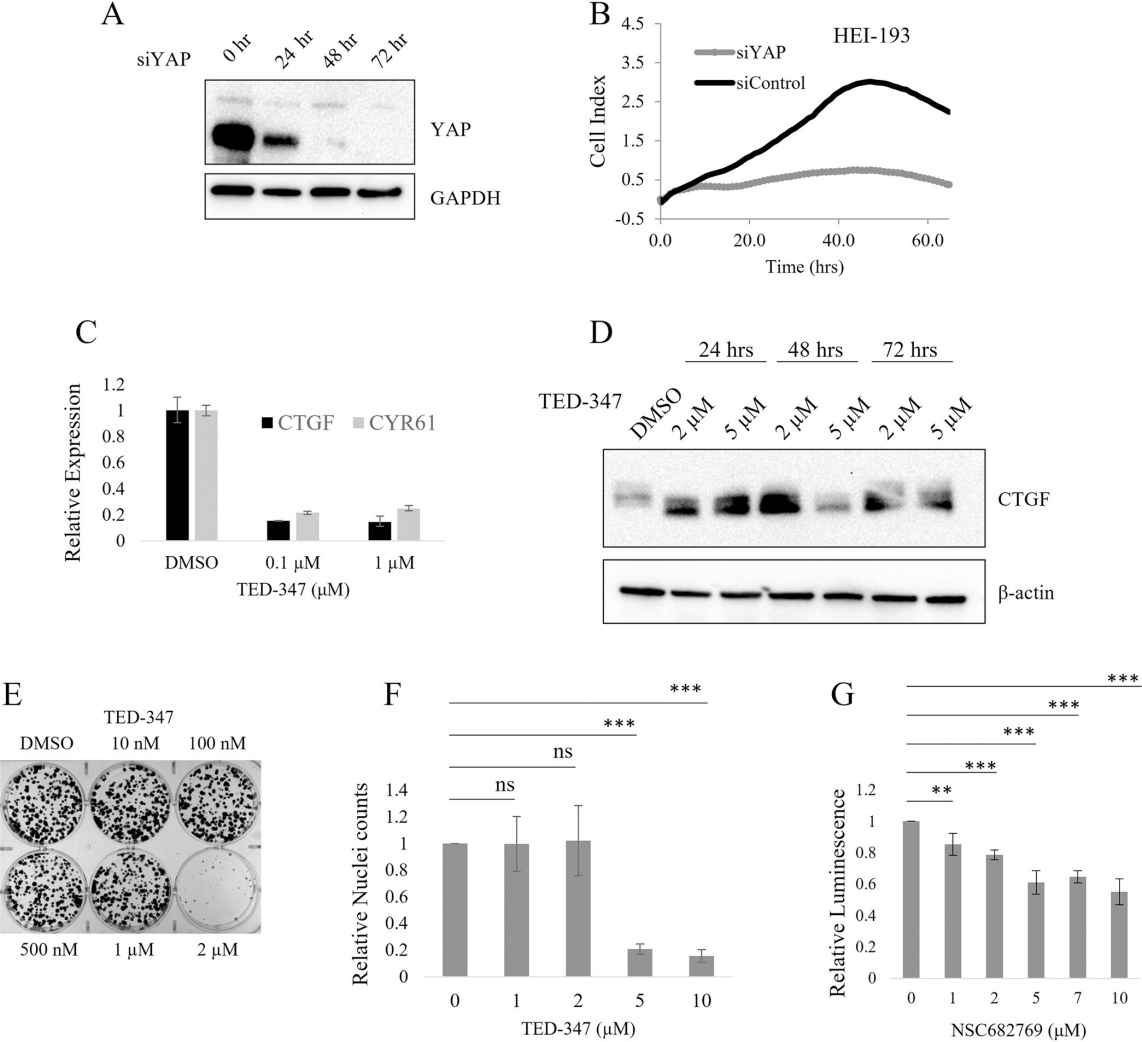
Genetic and pharmacological YAP-TEAD inhibition prevents cell growth of human NF2-deficient schwannoma. Immunoblot analysis confirms reduced expression of YAP after siYAP transfection (A). Real-time cell proliferation after siYAP or siControl transfection was monitored using Xcelligence RTCA system (B). Expression of Hippo pathway targets CTGF and CYR61 in HEI-193 cells after TED-347 treatment for 48 hours was determined by qRT-PCR (C). Immunoblot analysis confirms reduced CTGF and CYR61 protein levels after TED-347 treatment for 24, 48, or 72 hours (D). Colony formation assays were performed to assess the efficacy of TED-347 (E). Nuclei counts were performed on HEI-193 cells treated with TED-347 (F). Cell viability was determined by CellTiter-Glo for HEI-193 cells treated with NSC682769 for 72 hours (G). Error bars depict SD. ns = not significant; ** = p<0.01; *** = p<0.001.

Genetic ablation of YAP, the downstream effector of the Hippo pathway, by siRNA in human schwannoma cells prevented growth (Fig 2A and 2B), suggesting that small molecule inhibitors of this pathway might be beneficial. Because YAP lacks a DNA binding domain, binding to one of the TEAD family proteins within the nucleus is required to promote transcription of Hippo target genes [10]. YAP and TEAD binding occurs via a large, flat surface which is difficult to target with small molecules as there are no obvious binding pockets [18]. However, recent efforts have been successful by targeting TEAD palmitoylation binding sites, which are essential for YAP-TEAD binding [19]. The YAP-TEAD inhibitor TED-347 binds to a conserved cysteine within the palmitoylation pocket and prevents transcription of Hippo target genes such as Connective tissue growth factor (CTGF) and Cysteine-rich angiogenic inducer 61 (CYR61) at both mRNA and protein levels (Fig 2C and 2D). At low micromolar doses, TED-347 prevented colony formation of HEI-193 cells (Fig 2E). At 72 hours, TED-347 significantly reduces number ot HEI-193 cells, measured by nuclei count (Fig 2F). Another TEAD inhibitor, NSC682769, has been successful in models of glioblastoma [20]. This drug was moderately successful in reduction of human schwannoma HEI-193 cells over 72 hours (Fig 2G).

**Fig 3:**
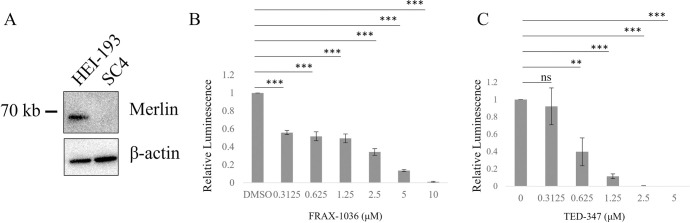
Group I PAK and YAP-TEAD inhibition reduces cell growth in mouse NF2-deficient schwannoma. Immunoblot analysis confirms truncated (HEI-193) and absence (SC4) of Merlin protein in schwannoma cell lines (A). SC4 cells in 0.5% FBS media were treated with FRAX-1036 for 7 days and cell viability was determined by CellTiter-Glo (B). SC4 cells in 0.5% FBS media were treated with TED-347 for 7 days and cell viability was determined by CellTiter-Glo (C). Error bars depict SD. ns = not significant; ** = p<0.01; *** = p<0.001.

HEI-193 is a human schwannoma cell line that contains a splice-site mutation that produces a short (and inactive) version of the Merlin protein. The mouse schwannoma line SC4, on the other hand, produces no Merlin protein (Fig 3A). As with HEI-193 cells, we found that the Group I PAK inhibitor FRAX-1036 and the TEAD inhibitor TED-347, respectively, reduced viability of SC4 cells (Fig 3B and 3C). However, unlike HEI-193 cells, SC4 cells required serum to be reduced to 0.5% FBS and drug incubation to be extended to 7 days before cell viability was reduced. Due to this limitation, HEI-193 cells were selected for future experiments.

**Fig 4:**
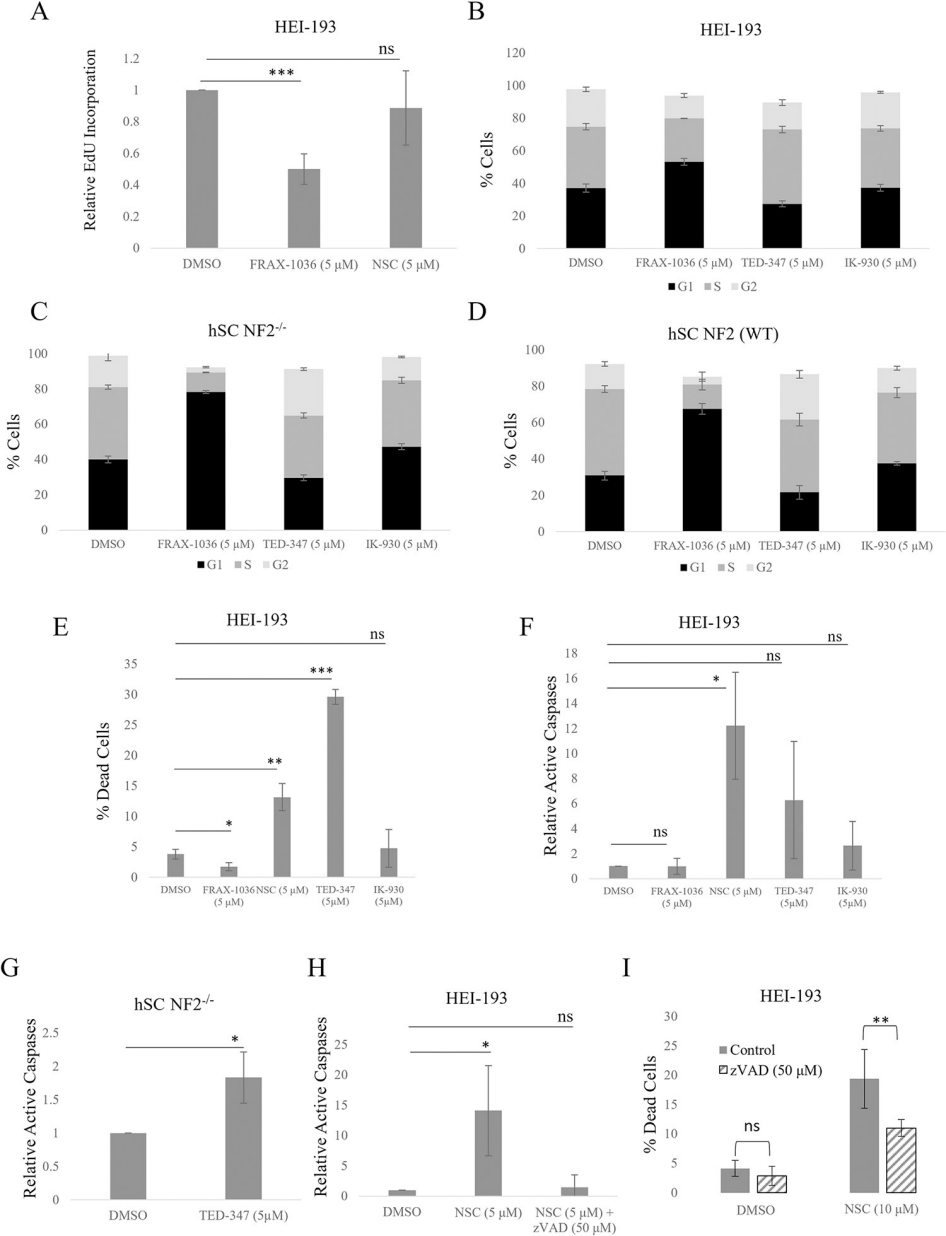
PAK inhibition blocks proliferation while YAP-TEAD inhibition promotes apoptosis. HEI-193 cells were treated with indicated drugs for 24 hours and proliferation was determined by EdU incorporation (A). Cell cycle was analyzed by flow cytometry after being treated with indicated drugs for 24 hours (B). Cells were treated with indicated drugs for 24 hours and simultaneously labeled with green-fluorescent calcein-AM (live) and red-fluorescent ethidium homodimer-1 (dead) and dead cells were quantified by flow cytometry (E). HEI-193 or hSC NF2-null cells were treated with indicated drugs and active caspases were labeled with TF2-VAD-FMK and cells were quantified by flow cytometry (F, G, H). Cells were treated with indicated drugs for 24 hours and simultaneously labeled with green-fluorescent calcein-AM (live) and red-fluorescent ethidium homodimer-1 (dead) and dead cells were quantified by flow cytometry (I). Error bars depict SD. ns = not significant; * = p<0.05; ** = p<0.01; *** = p<0.001.

To determine the effect of Group I PAK or TEAD inhibition and on cell proliferation, we evaluated EdU incorporation in HEI-193 cells after 48 hours of FRAX-1036 or TEAD inhibitor NSC682769 treatment (Fig 4A). The PAK inhibitor FRAX-1036 reduced EdU incorporation whereas the TEAD inhibitor NSC682769 had no effect. DNA content was labeled with propidium iodide to determine cell cycle. After 24 hours of treatment, FRAX-1036-treated human HEI-193 schwannoma cells had significantly more cells in G1 phase while TED-347-treated cells showed little difference from control (Fig 4B). We tested FRAX-1036 in a pair of human Schwann cell (hSC) lines, one of which has been made NF2-null via CRISPR [15]. In both NF2-null and wildtype cells, FRAX-1036 resulted in an increase in cells in the G1 phase (Fig 4C and 4D).

Human schwannoma HEI-193 cells treated with TEAD inhibitors NSC682769 and TED-347 had a larger dead cell population (calcein-AM positive, Ethidium Homodimer negative) after 72 hours of treatment compared to control. However, IK-930 treated cells showed no significant increase in the dead cell population. Group I PAK inhibitor treated cells showed a small decrease in the percentage of dead cells (Fig 4E). To determine the cause of cell death, cells were treated with FRAX-1036 or the TEAD inhibitors for 72 hours, stained for active caspases with TF2-VAD-FMK, and evaluated by flow cytometry. Cells treated with TEAD inhibitor NSC682769 had greater than 10-fold increase in active caspases compared to control cells. Cells treated with TEAD inhibitors TED-347 and IK-930 showed increased active caspases, but this did not reach statistical significance. The Group I PAK inhibitor had no effect (Fig 4F). NF2-null hSC cells treated with TED-347 had increased levels of active caspases (Fig 4G) Increased active caspases and dead cell populations in NSC682769 treated cells were abrogated by a caspase inhibitor (Fig 4H–4I), consistent with an apoptotic mechanism of action for the TEAD inhibitor.

**Fig 5:**
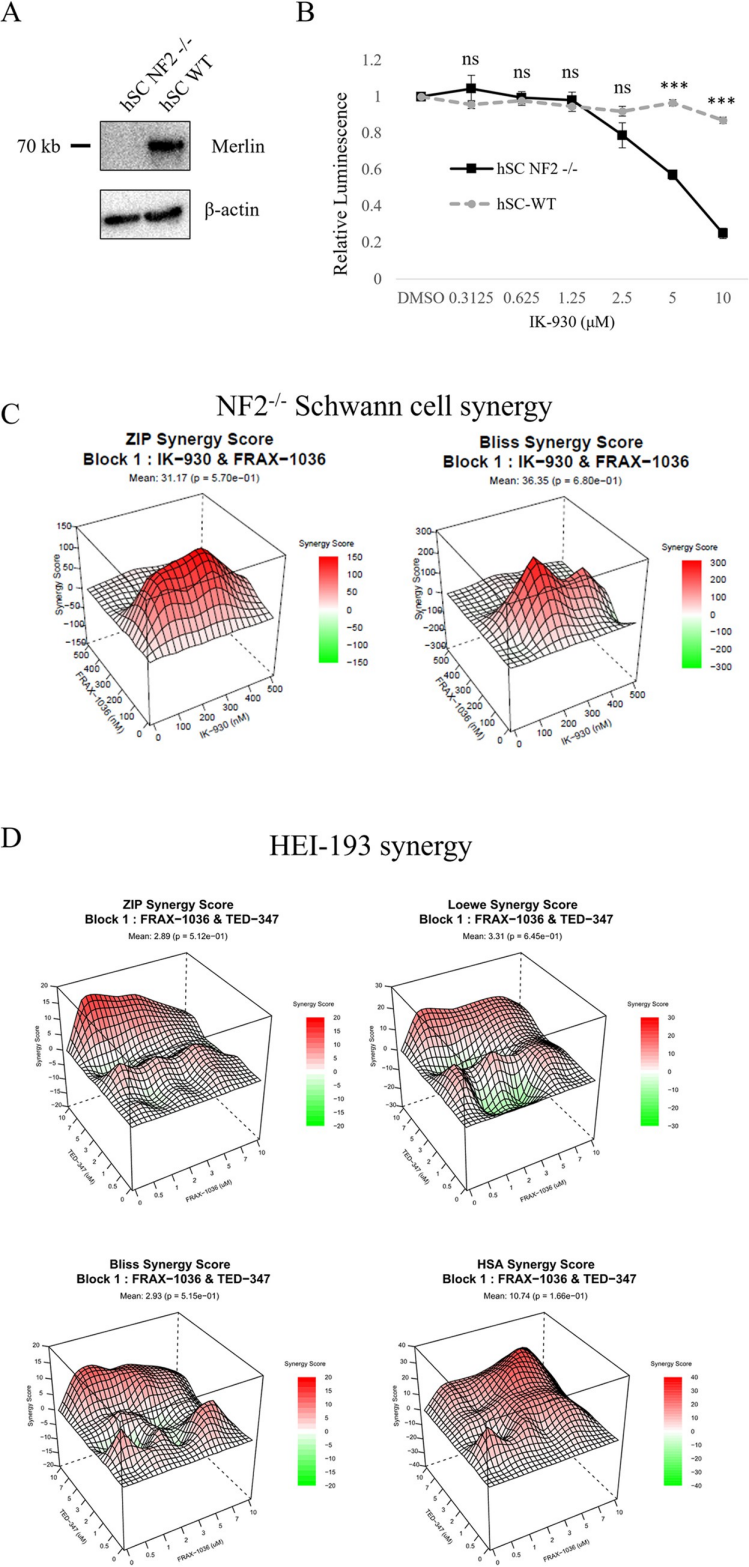
Simultaneous Group I PAK and YAP-TEAD inhibition produces a synergistic effect. Immunoblot shows Merlin protein levels in matched human Schwann cell ines (A). Merlin null and WT matched human Schwann cells were treated with TEAD palmitoylation inhibitor IK-930 (B). NF2-null human Schwann cells were treated with increasing concentrations of IK-930 and FRAX-1036 for 5 days and synergy scores were calculated (C). HEI-193 cells were treated with increasing concentrations of TED-347 and FRAX-1036 and synergy scores were calculated (D). Error bars depict SD. ns = not significant; ** = p<0.01; *** = p<0.001.

Despite mixed results between various TEAD inhibitors we decided to further investigate IK-930, which has already been utilized in clinical trials (NCT05228015), because TED-347 and NSC682769 would not be viable for *in vivo* studies or clinical applications. (Fig 5A). IK-930 reduced cell growth at micromolar concentrations in the NF2-null human Schwann cells but not their wildtype counterparts (Fig 5B). To determine the efficacy of a TEAD and group I PAK inhibitor combination treatment, we treated both HEI-193 cells and hSC NF2-null cells with increasing concentrations of both drugs. Viability was measured using CellTiter-Glo and synergy was determined using the SynergyFinder+ web application [21]. Typically, synergy scores above 10 indicate synergy. NF2-null human Schwann cells had large peaks of synergy scores with a mean score over 30 for both Zero interaction potency (ZIP) and Bliss synergy models (Fig 5C). HEI-193 cells also had significant peaks of synergy scores, but only the highest single agent (HSA) model had a mean above 10 (Fig 5D).

## Discussion

In this study, we explored the possibility of treating NF2-deficient schwannomas with Group I PAK inhibitors, TEAD inhibitors, or combinations of the two. NF2 syndrome has been difficult to target due to its slow-growing nature. Each mechanism-based new pharmacotherapy proposed to date has shown at best partial efficacy in a subset of patients, accompanied by serious toxicities. The most successful therapy to date has been the anti-VEGF drug bevacizumab. However, there are long term concerns for patients who start bevacizumab during adolescence, so many pediatric NF2 patients, who often have the most severe form of the disease, are restricted to surgical treatment. Even older patients taking bevacizumab often experience hypertension and proteinuria, requiring frequent treatment breaks [22]. Many NF2 patients are enrolled in clinical drug trials but often still need to undergo complicated and invasive surgery to preserve hearing and relieve brainstem compression.

NF2 syndrome is characterized by the loss or severe truncation of Merlin protein. Merlin plays multiple roles in the cell, including inhibition of PAK1. With loss of Merlin due to NF2 syndrome, PAK1 is overactivated promoting excess proliferation. We and others have previously shown that NF2-deficient schwannomas and meningiomas responds to PAK inhibitors *in vivo* [5,6]. In addition, PAK1 specific inhibition has been shown to be moderately effective in reducing tumor size and increasing survival of a genetically engineered mouse model of NF2 [17]. Interestingly, genetic deletion of *Pak1*, but not *Pak2*, in this model had partial beneficial effects on tumor growth and on hearing preservation, suggesting that PAK1 alone might prove a reasonably therapeutic for NF2-deficient malignancies, thus avoiding the cardiotoxicities associated with PAK2 inhibition. However, the studies and by presented here and by Licciulli *et al*. suggest that Group I PAKs play at least partly redundant proliferative signaling roles in NF2-deficient cells and thus a non-isoform selective Group I PAK inhibitor may be required.

Another role of Merlin involves activation of the Hippo pathway. Merlin’s scaffolding functions promote the phosphorylation and activation of the Hippo kinase cascade, which acts as a tumor suppressor by phosphorylating YAP and targeting it for degradation. Without Merlin, YAP is translocated to the nucleus, binds to the TEAD transcription factors and promotes proliferation and anti-apoptotic target genes. Targeting the Hippo pathway has recently become more plausible due to the development of TEAD palmitoylation inhibitors that block YAP-TEAD binding and prevent transcription of target genes. In early clinical trials, these drugs, along with inhibitors that directly block YAP-TEAD interactions, have been found to be well tolerated and show antitumor activity in NF2-mutant cancers and mesothelioma, a condition known to often have NF2 alterations (NCT04665206, NCT05228015, NCT04857372) [23].

While the recent success of TEAD inhibitors in other NF2-altered cancers is exciting, the slow-growing, benign nature of schwannomas may make them difficult to target with any monotherapy. Combination therapy can reduce negative side effects or off target effects of drugs by requiring lower doses of each individual drug while achieving the same effect. Recent work has identified combined PI3K and PAK inhibition which reduces viability and promotes apoptosis in cellular models of NF2 as well as *in vivo* [24]. In this study, we confirm previous work that shows group I PAK inhibition successfully reduces cell growth of the schwannoma cell lines HEI-193 and SC4. Due to the cardiotoxicities associated with PAK2 inhibition, recent developments in PAK1-specific drugs have been of interest for Merlin-deficient schwannomas. However, we found that the PAK1-specific inhibitor NVS-PAK1-1 was less efficient in blocking cell growth compared to two Group I PAK inhibitors, FRAX-1036 and G-5555 (Fig 1C and 1D). In an attempt to find additional agents that might be combined with PAK inhibitors, we also evaluated three different recently developed TEAD inhibitors, which target the Hippo pathway by blocking TEAD palmitate binding, restricting TEAD-mediated transcription. We showed that genetic ablation of YAP blocks cell growth in HEI-193 cells (Fig 2) and that TEAD inhibitors are effective at reducing Hippo target gene expression and in reducing cell number of NF2-deficient schwannoma cells at 72 hours post treatment. Similar results were obtained in another cellular model of Merlin-deficient schwannoma, SC4, a mouse schwannoma cell line that produces no Merlin protein (Fig 3).

TEAD and PAK inhibitors affect schwannoma cells in distinct ways. as demonstrated by their effects on proliferation and apoptosis. Cells treated with FRAX-1036 for 72 hours had reduced populations of proliferating cells, measured by EdU incorporation, while cells treated with TEAD inhibitor NSC682769 were unchanged. Cells treated with FRAX-1036 had a change in cell cycle profile with an increase in cells in the G1 phase while cells treated with TED-347 were unchanged from control. These data suggest that Group I PAK inhibition disrupts the cell cycle and prevents proliferation in Merlin-deficient schwannoma cells. This data is consistent with Mercado-Pimentel and colleagues’ work with PAK inhibitors PI-8 and PI-15 which showed that PAK inhibition induced mitotic catastrophe but not apoptosis in HEI-193 cells [25]. A similar Group I PAK inhibitor FRAX-597 reduces proliferation and increases the G1 cell population in SC4 cells [6]. On the other hand, cells treated with TEAD inhibitor TED-347 had a larger pool of dead cells compared to FRAX-1036 treated or control cells, showing a 12-fold increase in generic active caspases (Fig 4). Taken together, these data suggest that, in Merlin-deficient schwannoma cells, Group I PAK inhibition blocks proliferation and TEAD inhibition promotes cell death, specifically apoptosis, suggesting a rationale for combining these agents.

Given the large difference between the inhibitor concentration needed to reduce mRNA levels of TEAD targets CTGF and CYR61 and the concentration needed to reduce cell number and promote cell death, it is likely that other factors may be influencing cell survival. YAP-TEAD binding promotes transcription of many varied targets. While CTGF and CYR61 are the most popular targets to confirm inhibition of TEAD, other targets may require higher concentrations of drug and may be interacting with other proteins both within and outside the Hippo pathway. The exact mechanism of reduced proliferation and promotion of apoptosis would be an interesting future topic of study.

When we combined TEAD inhibitors IK-930 or TED-347 with FRAX-1036, we found a synergistic effect in two different Merlin-deficient cell lines using several different models to predict synergy (Fig 5). The HSA synergy model is based on the assumption that if a drug combination has a higher effect than its constituent parts, there must be a synergistic interaction [26]. This model is useful for determining synergy of chemicals with unrelated targets. The ZIP model assumes that two non-interacting drugs incur minimal changes in their dose–response curves [27]. Synergy between drugs with non-logistic dose-response patterns will not be accurately reported by the ZIP model. The Bliss independence model assumes a stochastic process in which two drugs elicit their effects (statistically) independent of one another. The Loewe model compares the observed effect of the compounds to an additive effect that assumes the two drugs are the same compound [28]. Human schwannoma HEI-193 cells only showed average synergy scores above 10 in the HSA model. However, considering the relatively high doses of TEAD inhibitor TED-347 needed to reduce cell viability in this model, it is not surprising that synergy only occurs when high amounts of drug are present.

HEI-193 cells have been a popular model for NF2-related schwannoma and were therefore included in many parts of this study. However, others have shown that the truncated version of Merlin still retains moderately active tumor suppressive abilities [29]. HEI-193 cells, therefore, may not be a good model for most NF2 patient tumors. A new cell line from a patient with a sporadic vestibular schwannoma has recently been created [30]. However, this cell line also produces some Merlin protein, albeit less than HEI-193 cells and much less than wild-type Schwann cells. Until a new NF2-null human cell line has been created, it may be helpful to test potential drugs in a truly NF2-null environment, such as the NF2 knock-out human Schwann cells used in this study.

In summary, we demonstrated that in NF2-deficient schwannoma, TEAD inhibition and Group I PAK inhibition are successful in preventing cell growth. TEAD inhibition promotes cell death while PAK inhibition restricts proliferation. The combination of these drugs promotes a synergistic effect. Combination therapy involving these two targets may be beneficial for NF2 patients and further research into these targets and others should consider combination therapy as a way to increase efficacy in this difficult to treat disease.

## Supporting information

S1 Dataset (XLSX)

S1 Raw images (PDF)
