# Supplementary material for: Synergistic effect of PAK and Hippo pathway inhibitor combination in NF2-deficient Schwannoma
Source: PLoS One. 2024 Jul 31;19(7):e0305121. doi: 10.1371/journal.pone.0305121 (PMC11290668; doi:10.1371/journal.pone.0305121)

Figure 1E, 4 hours: Total PAK

Captured on FluorChem E System

Lane 1: Ladder

Lane 2: DMSO

Lane 3: 2µ FRAX-1036

Lane 4: 5µ FRAX-1036

Lane 5: 2µ NVS-PAK-1-1

Lane 6: 5µ NVS-PAK-1-1

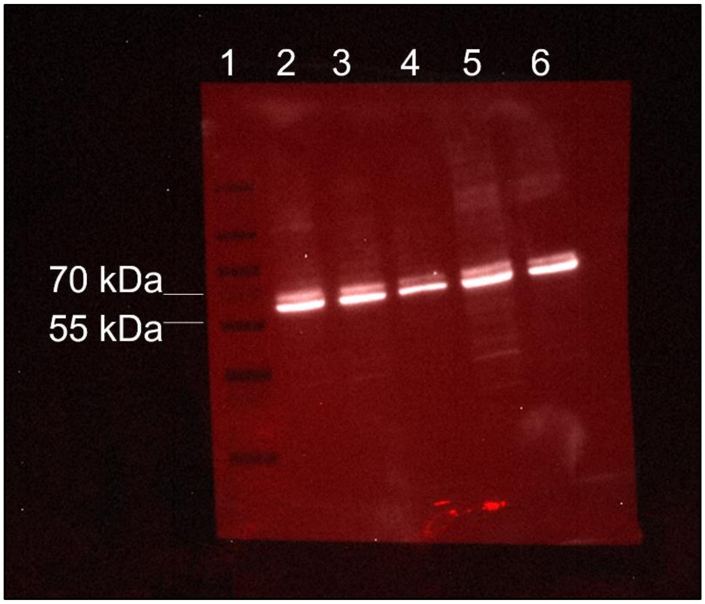

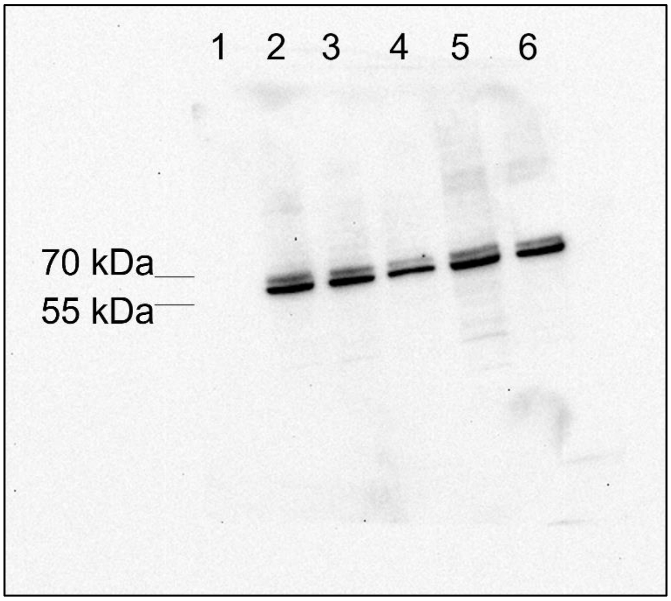

Figure 1E, 4 hours: GAPDH

Captured on FluorChem E System

Lane 1: Ladder

Lane 2: DMSO

Lane 3: 2µ FRAX-1036

Lane 4: 5µ FRAX-1036

Lane 5: 2µ NVS-PAK-1-1

Lane 6: 5µ NVS-PAK-1-1

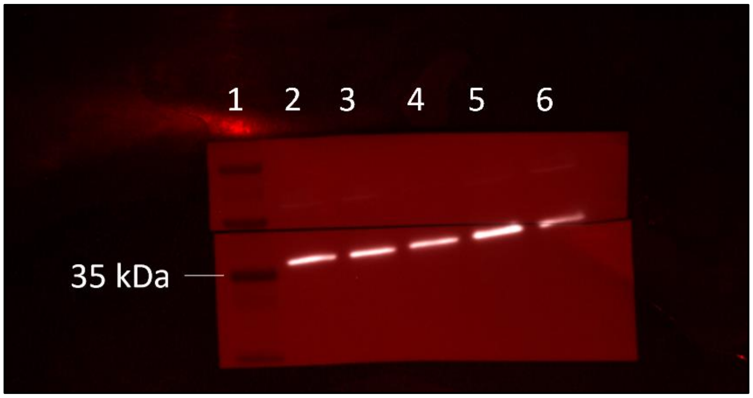

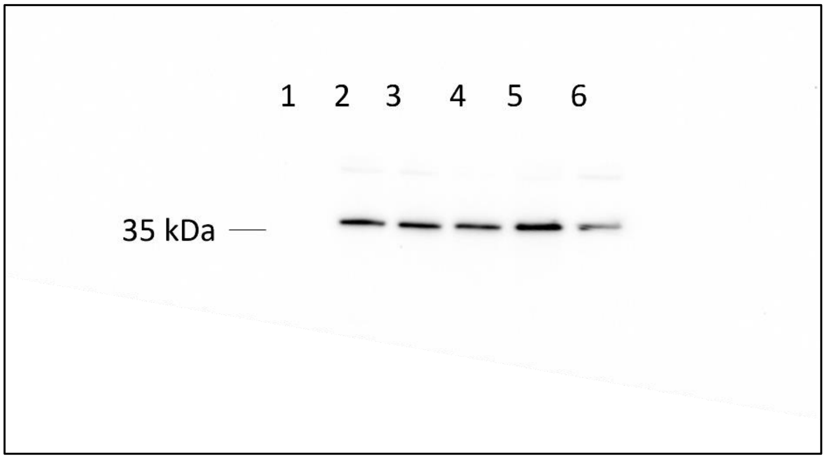

Figure 1E, 4 hours, Phospho-PAK

Captured on FluorChem E System

Lane 1: Ladder

Lane 2: DMSO

Lane 3: 2μ FRAX-1036

Lane 4: 5μ FRAX-1036

Lane 5: 2μ NVS-PAK-1-1

Lane 6: 5μ NVS-PAK-1-1

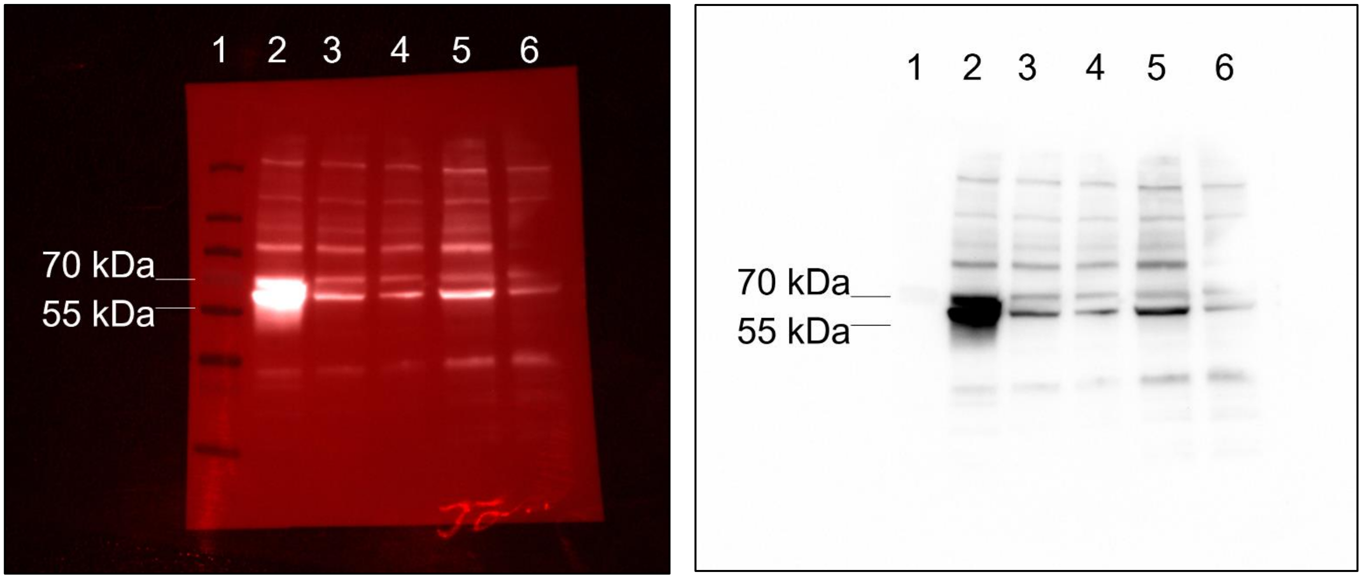

Figure 1E, 4 hours , GAPDJH

Captured on FluorChem E System

Lane 1: Ladder

Lane 2: DMSO

Lane 3: 2µ FRAX-1036

Lane 4: 5µ FRAX-1036

Lane 5: 2µ NVS-PAK-1-1

Lane 6: 5µ NVS-PAK-1-1

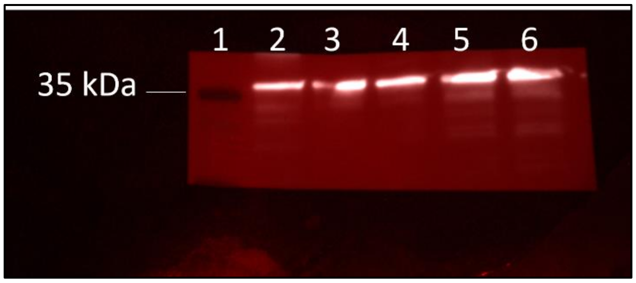

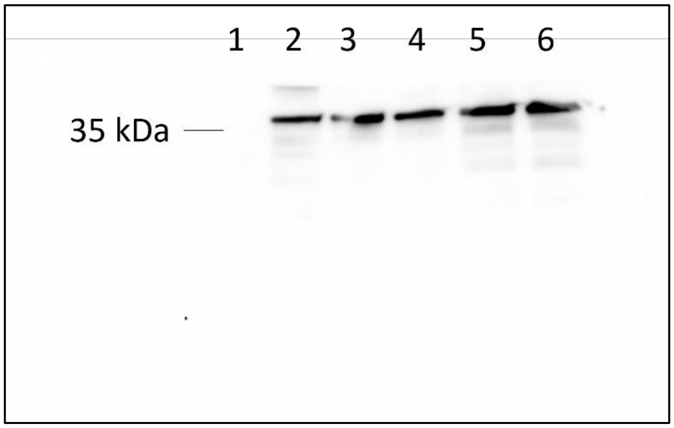

Figure 1E, 72 hours , Total PAK

Captured on FluorChem E System

Lane 1: Ladder

Lane 2: DMSO

Lane 3: 1 μ NVS-PAK-1-1

Lane 4: 2 μ NVS-PAK-1-1

Lane 5: 5 μ NVS-PAK-1-1

Lane 6: 10 μ NVS-PAK-1-1

Lane 7: Ladder

Lane 8: DMSO

Lane 9: 1μ FRAX-1036

Lane 10: 2μ FRAX-1036

Lane 11: 5μ FRAX-1036

Lane 12: 10μ FRAX-1036

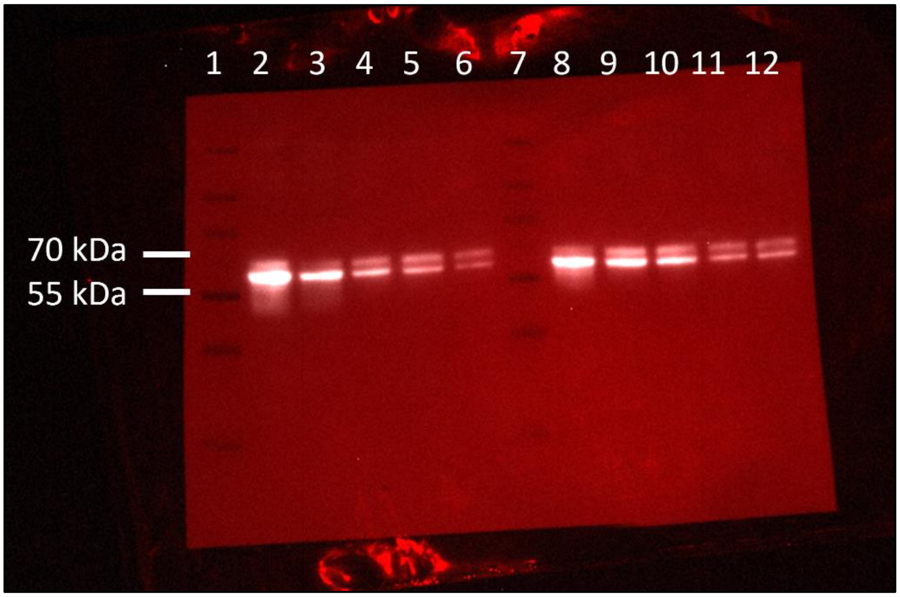

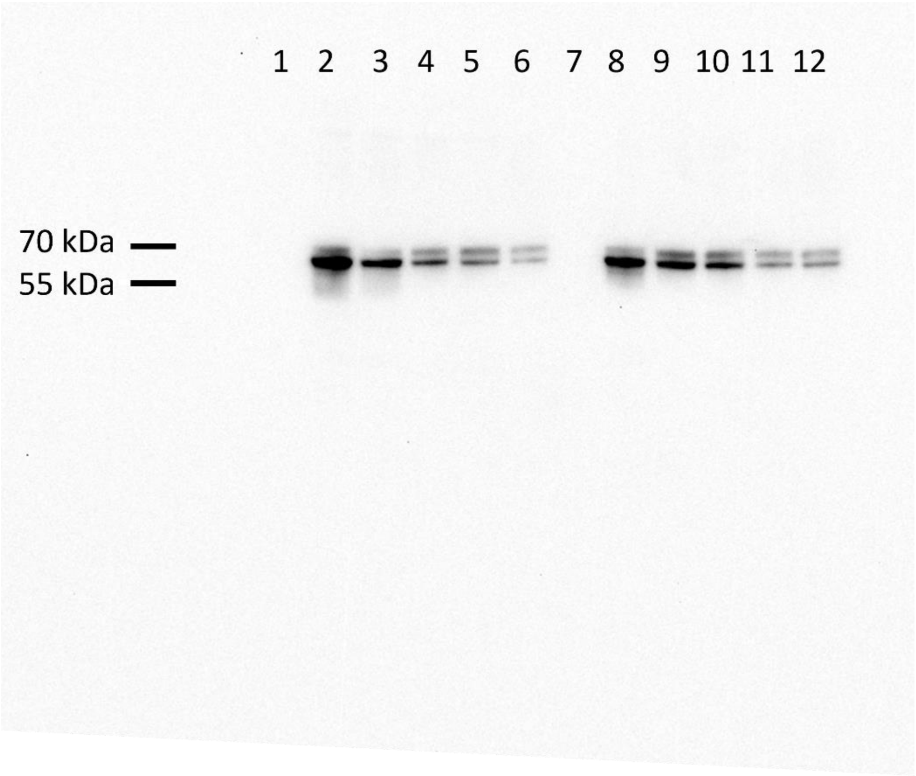

Figure 1E, 72 hours , Phospho-PAK

Captured on FluorChem E System

Lane 1: Ladder

Lane 2: DMSO

Lane 3: 1 μ NVS-PAK-1-1

Lane 4: 2 μ NVS-PAK-1-1

Lane 5: 5 μ NVS-PAK-1-1

Lane 6: 10 μ NVS-PAK-1-1

Lane 7: Ladder

Lane 8: DMSO

Lane 9: 1μ FRAX-1036

Lane 10: 2μ FRAX-1036

Lane 11: 5μ FRAX-1036

Lane 12: 10μ FRAX-1036

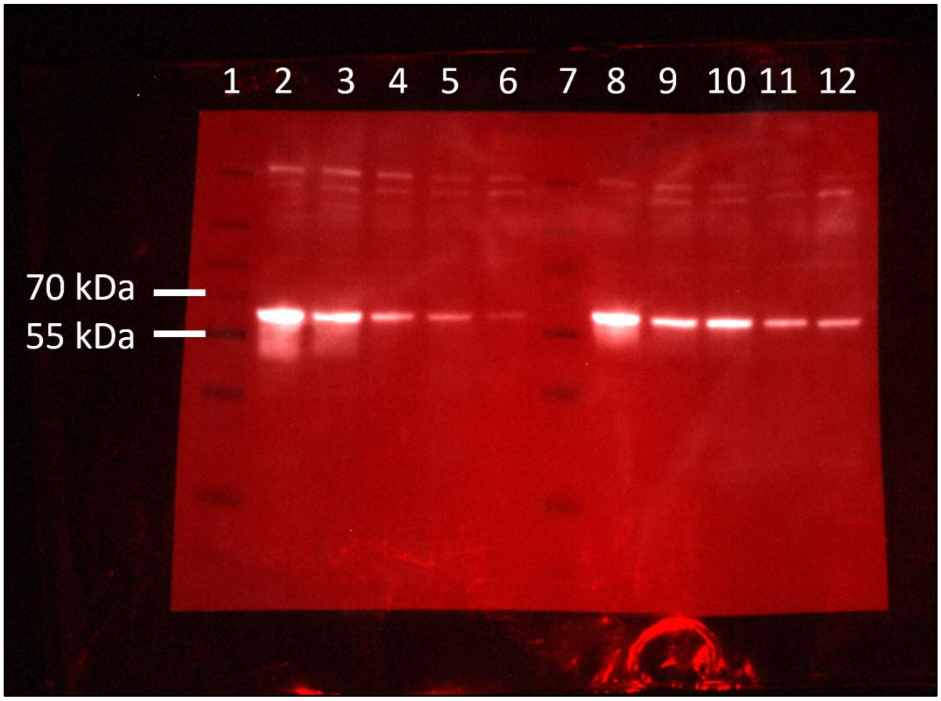

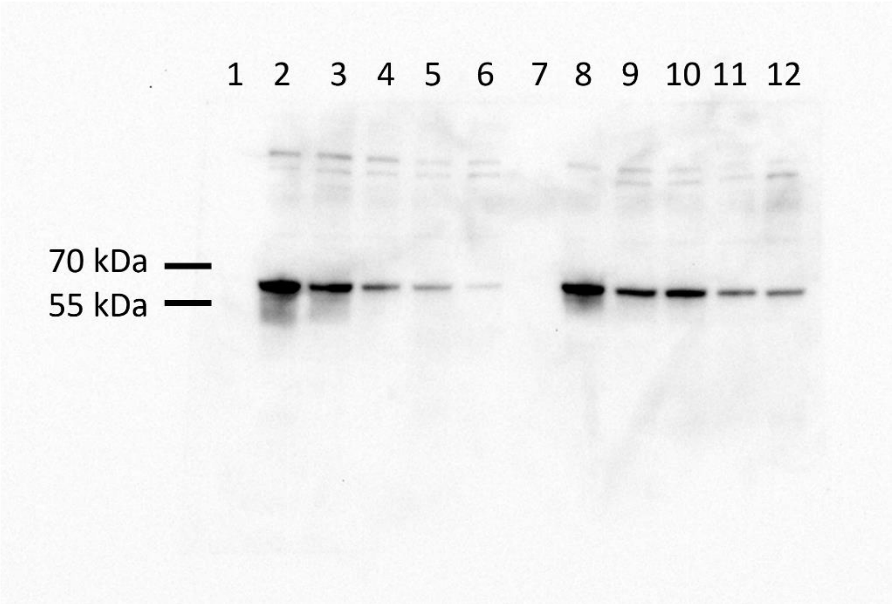

Figure 1E, 72 hours , GAPDH

Captured on FluorChem E System

Lane 1: Ladder

Lane 2: DMSO

Lane 3: 1 µ NVS-PAK-1-1

Lane 4: 2 µ NVS-PAK-1-1

Lane 5: 5 µ NVS-PAK-1-1

Lane 6: 10 µ NVS-PAK-1-1

Lane 7: Ladder

Lane 8: DMSO

Lane 9: 1µ FRAX-1036

Lane 10: 2µ FRAX-1036

Lane 11: 5µ FRAX-1036

Lane 12: 10µ FRAX-1036

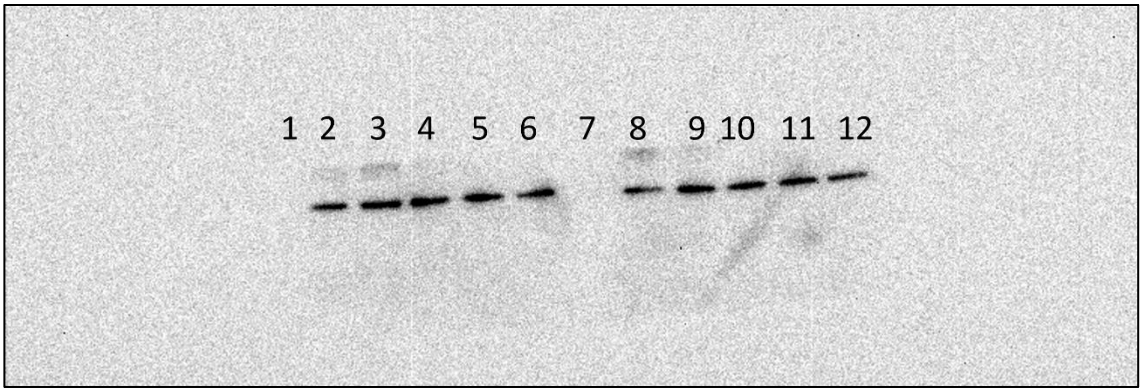

Figure 2A, YAP

Captured on FluorChem E System

Lane 1: Ladder

Lane 2: HEI-193, siYAP, 0hr

Lane 3: HEI-193, siYAP, 24hr

Lane 4: HEI-193, siYAP, 48hr

Lane 5: HEI-193, siYAP, 72hr

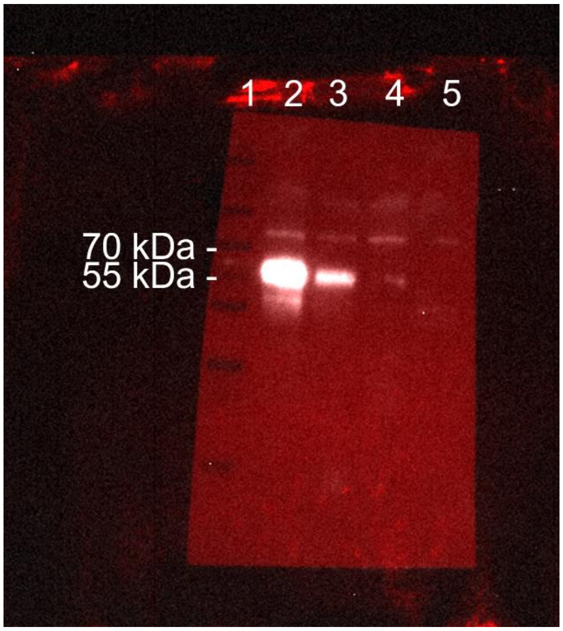

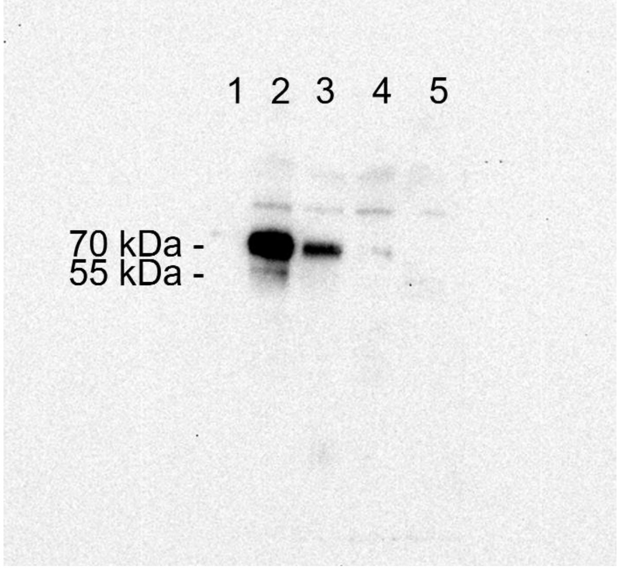

Figure 2A, GAPDH

Captured on FluorChem E System

Lane 1: Ladder

Lane 2: HEI-193, siYAP, 0hr

Lane 3: HEI-193, siYAP, 24hr

Lane 4: HEI-193, siYAP, 48hr

Lane 5: HEI-193, siYAP, 72hr

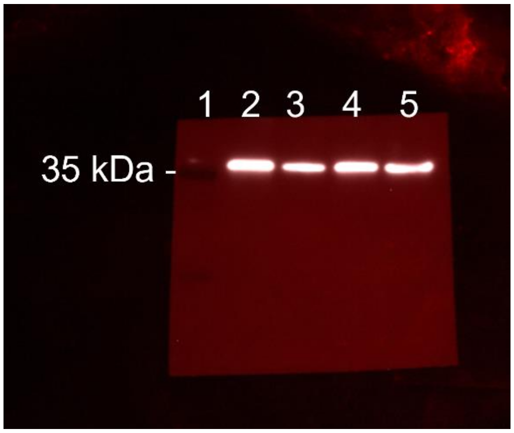

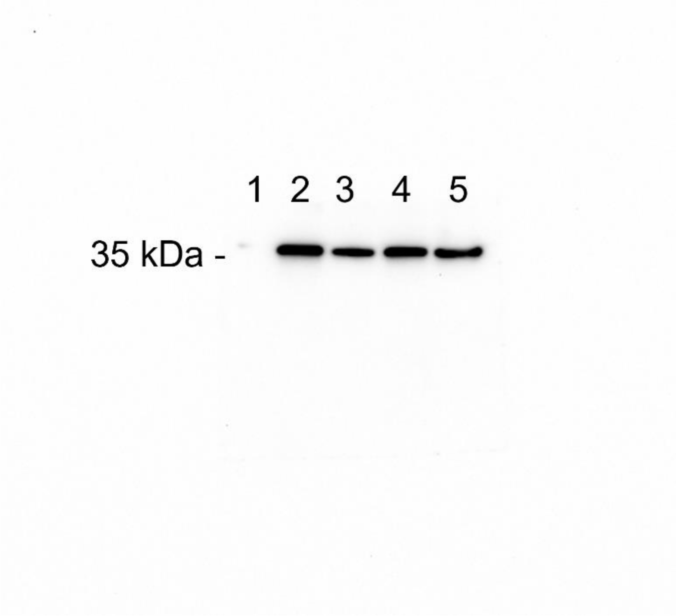

Figure 2D, CTGF

Captured on FluorChem E System

Lane 1: Ladder

Lane 2: HEI-193, DMSO

Lane 3: HEI-193, TED-347, 2μM, 24 hrs

Lane 4: HEI-193, TED-347, 5μM, 24 hrs

Lane 5: HEI-193, TED-347, 2μM, 48 hrs

Lane 6: HEI-193, TED-347, 5μM, 48 hrs

Lane 7: HEI-193, TED-347, 2μM, 72 hrs

Lane 8: HEI-193, TED-347, 5μM, 72 hrs

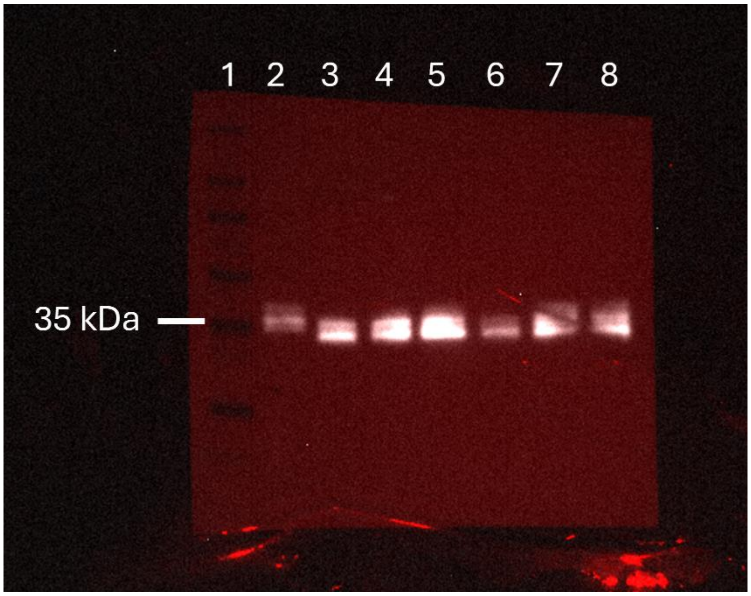

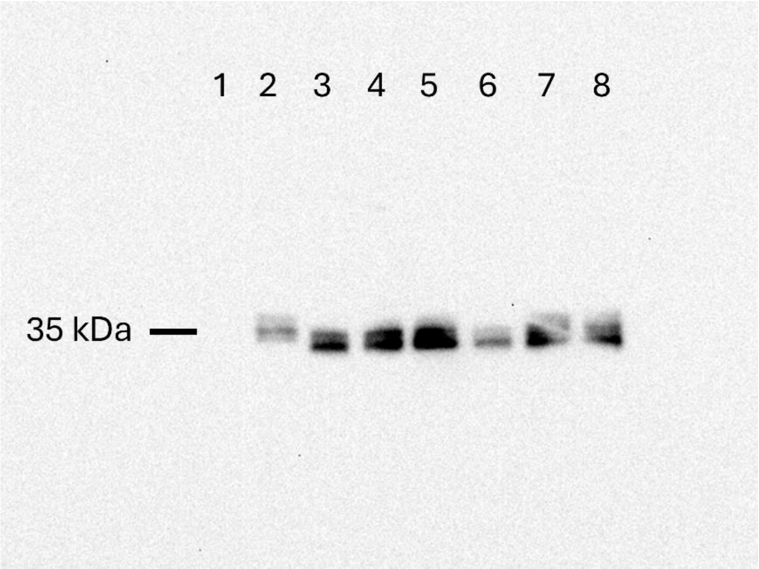

Figure 2D, β-actin

Captured on FluorChem E System

Lane 1: Ladder

Lane 2: HEI-193, DMSO

Lane 3: HEI-193, TED-347, 2μM, 24 hrs

Lane 4: HEI-193, TED-347, 5μM, 24 hrs

Lane 5: HEI-193, TED-347, 2μM, 48 hrs

Lane 6: HEI-193, TED-347, 5μM, 48 hrs

Lane 7: HEI-193, TED-347, 2μM, 72 hrs

Lane 8: HEI-193, TED-347, 5μM, 72 hrs

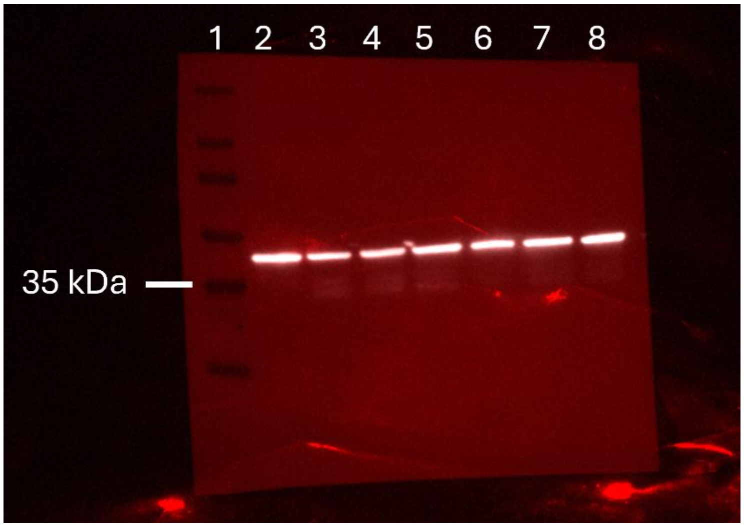

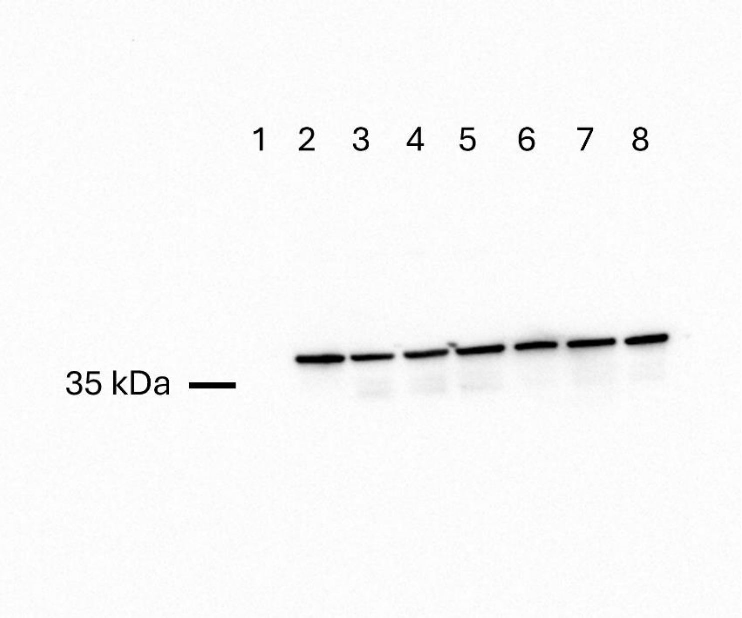

Figure 3A and 5A, Merlin

Captured on FluorChem E System

Lane 3: HEI-193 (Fig 3A)

Lane 4: SC4 (Fig 3A)

Lane 5: Human Schwann cells (NF2 null) (Fig 5A)

Lane 6: Human Schwann cells (NF2 wild-type) (Fig 5A)

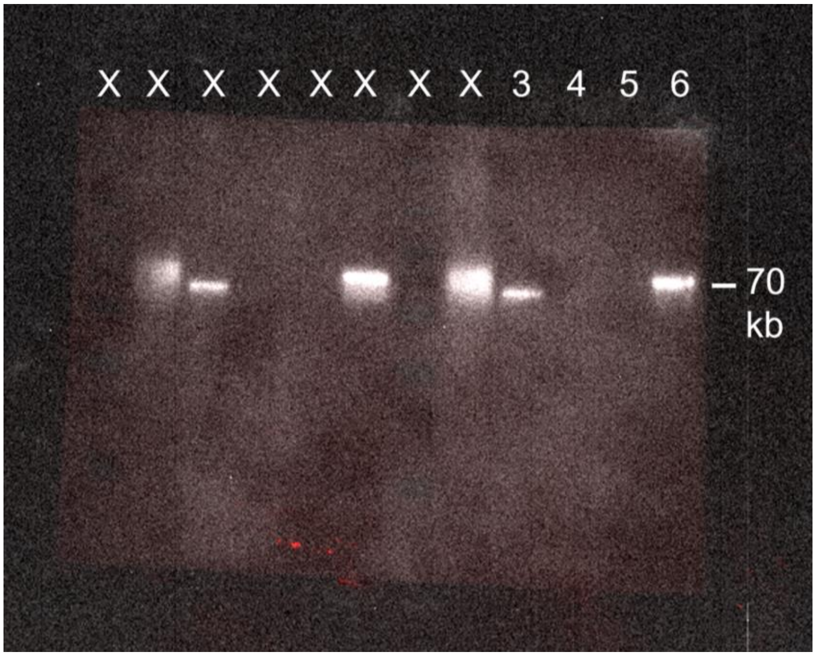

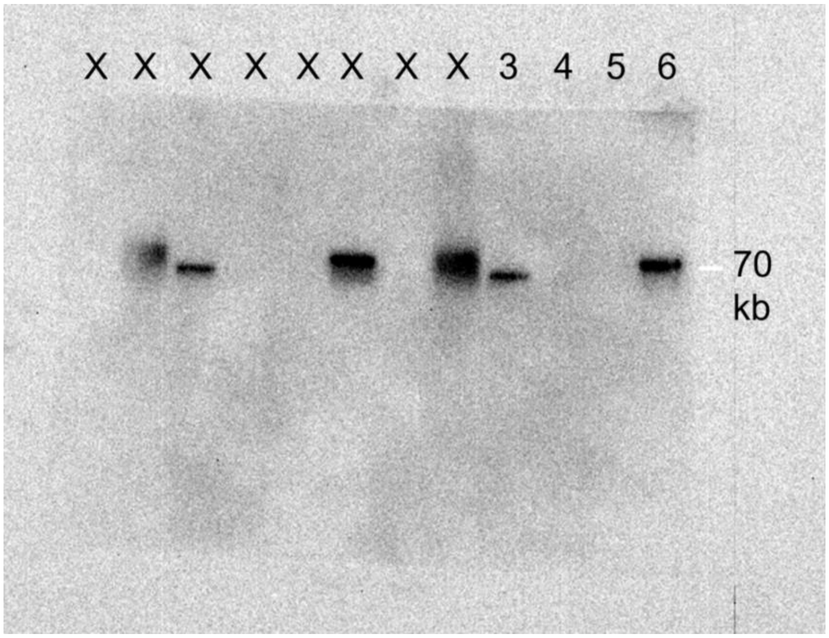

Figure 3A and 5A, β-actin

Captured on FluorChem E System

Lane 3: HEI-193 (Fig 3A)

Lane 4: SC4 (Fig 3A)

Lane 5: Human Schwann cells (NF2 null) (Fig 5A)

Lane 6: Human Schwann cells (NF2 wild-type) (Fig 5A)

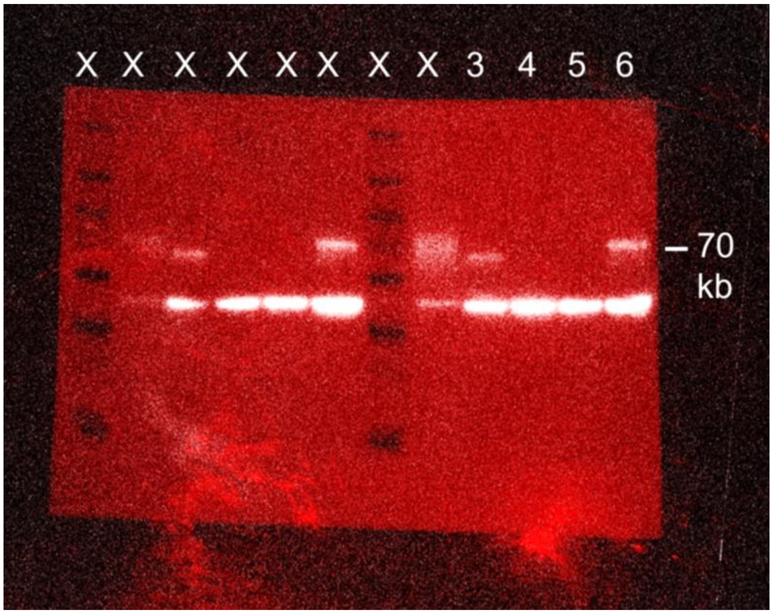

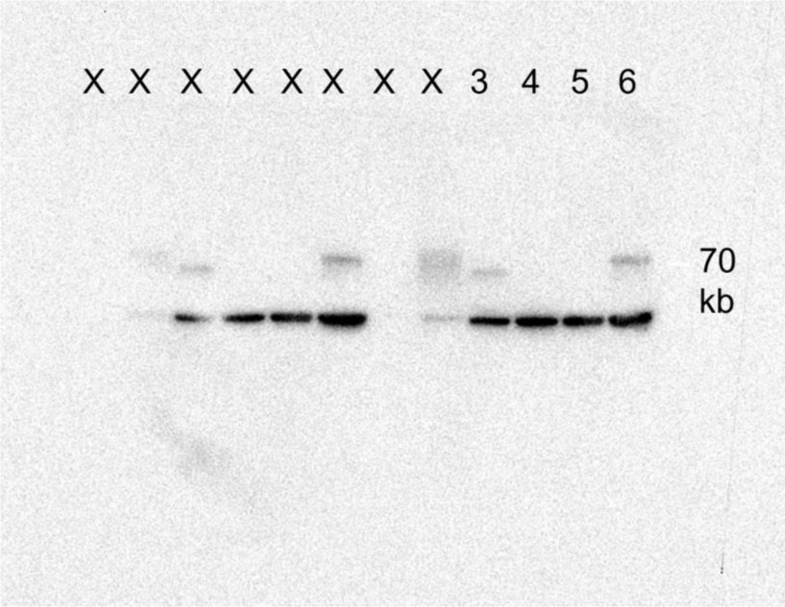

Supplement: S1 Raw images — (PDF) [file pone.0305121.s002.pdf]
